# Laparoscopic revision of open appendectomy complicated by high-output colo-cutaneous fistula: a case report

**DOI:** 10.1093/jscr/rjaf1022

**Published:** 2025-12-22

**Authors:** Ognen Kostovski, Daniel Jankoski, Irena Kostovska

**Affiliations:** University Clinic of Digestive Surgery, Clinical Center ‘Mother Theresa’ No. 17, Skopje, 1000, North Macedonia; University Clinic of Digestive Surgery, Clinical Center ‘Mother Theresa’ No. 17, Skopje, 1000, North Macedonia; Department of Medical and Experimental Biochemistry, Faculty of Medicine, Str. “50 Divizija” No. 6, Ss. Cyril and Methodius University in Skopje, Skopje, 1000, North Macedonia

**Keywords:** colo-cutaneous fistula, high-output fistula, laparoscopic appendectomy

## Abstract

Colo-cutaneous fistulae are rare complications following appendectomy, especially when high-output, and can cause significant morbidity. We report a 66-year-old male who presented nine days post-open appendectomy with fecal discharge from the incision. Imaging showed cecal adherence to the anterior abdominal wall, consistent with a colo-cutaneous fistula. Conservative management failed, with persistent high-output drainage of 600–1000 mL/day. The patient underwent laparoscopic revision, including adhesiolysis, mobilization of the terminal ileum and ascending colon, and resection with extracorporeal ileocolic anastomosis. Postoperative wound healing was managed with secondary intention and negative pressure therapy. The patient recovered well and was discharged in stable condition. This case demonstrates the effectiveness of laparoscopic intervention in high-output colo-cutaneous fistulae and highlights the need for timely surgical management when conservative measures fail.

## Introduction

Colo-cutaneous fistulae are rare but serious complications following appendectomy, with an incidence of less than 1% in most series. They represent abnormal communications between the colon and the skin, resulting in external discharge of intestinal contents [1]. These fistulae carry significant morbidity due to fluid and electrolyte loss, malnutrition, local infection, and impaired wound healing. High-output fistulae, defined as producing over 500 mL of output per day, pose particular challenges, being associated with higher rates of sepsis, prolonged hospitalization, and delayed recovery [2]. Etiology is multifactorial, commonly arising from unrecognized intraoperative bowel injury, anastomotic leak, abscess formation, or ischemia at the surgical site. Initial management typically involves conservative measures, including fluid and electrolyte replacement, nutritional support, wound care, and infection control. However, high-output or persistent fistulae often require definitive surgical intervention [3]. Minimally invasive approaches, particularly laparoscopy, provide enhanced visualization, precise adhesiolysis, reduced tissue trauma, and potentially faster recovery compared with open surgery [4]. Nevertheless, operative management remains technically demanding, requiring careful preoperative planning and advanced laparoscopic expertise. Given the rarity and complexity of post-appendectomy colo-cutaneous fistulae, especially high-output types, reporting such cases contributes valuable insights into surgical decision-making, perioperative management, and outcomes [5]. We present a case of a 66-year-old male with a high-output colo-cutaneous fistula following open appendectomy, ultimately requiring surgical intervention. Written informed consent was obtained for publication.

## Case presentation

**Figure 1:**
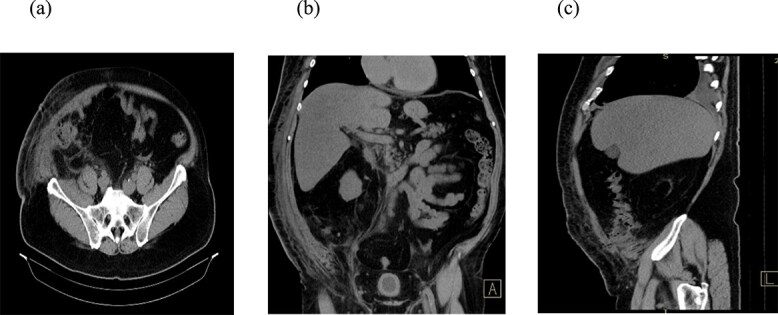
CT scan of the abdomen. Three reconstructions were evaluated: Axial (a), coronal (b), and sagittal (c). The findings suggest a collision of the cecum with the anterior abdominal wall at the site of the previous laparotomy incision, accompanied by surrounding mesenteric stranding. In the region of the anterior abdominal wall, there is fat stranding within the subcutaneous tissue, indicating inflammation, as well as air inclusions originating from the cecum, findings consistent with a colo-cutaneous fistula.

A 66-year-old patient was admitted to the Clinic for Digestive Surgery as an emergency case with a history of open appendectomy nine days earlier, operated on in another institution, with a classical appendectomy performed through a McBurney muscle-splitting incision. Upon admission, fecal content was observed in the area of the operative wound, raising suspicion of a colo-cutaneous fistula. On physical examination, the abdomen was soft and non-tender, without signs of peritoneal irritation. Fecal discharge was present at the site of the previous surgical incision. The patient was initially managed conservatively with dual antibiotic therapy, analgesics, antithrombotic medication, fluid and electrolyte replacement, wound care, and gastroprotective therapy. A collection device was applied to the wound, and the amount of fecal output was monitored. The patient had a high-output colo-cutaneous fistula, with daily drainage ranging from 600 to 1000 ml. Due to the failure of conservative management and the persistence of high-volume output, surgical intervention was indicated. Laboratory tests revealed mild electrolyte disturbances, leukocytosis (14.9 × 10^9^/L), and a C-reactive protein level of 86.3 mg/L. Imaging techniques, computed tomography (CT) scan of the abdomen (Fig. 1), demonstrated a collision of the cecum with the anterior abdominal wall at the site of the previous laparotomy incision, accompanied by surrounding mesenteric stranding. In the region of the anterior abdominal wall, fat stranding was found within the subcutaneous tissue, indicating inflammation, as well as air inclusions originating from the cecum, findings consistent with a colo-cutaneous fistula.

## Surgical procedure

**Figure 2:**
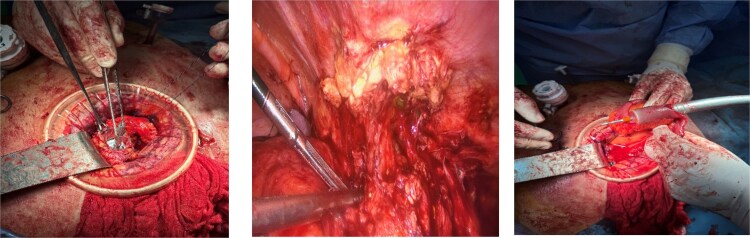
Surgical procedure.

The patient underwent laparoscopic revision. Working trocars were placed, and pneumoperitoneum was established. Intraoperatively, an inflammatory mass with adhesions to the anterior abdominal wall was identified at the previous laparotomy site. Careful adhesiolysis was performed, preserving viable tissue, and the cecum was freed from its attachment to the prior McBurney incision. The terminal ileum and ascending colon were mobilized. A relaparotomy was then performed through the previous incision, and a wound protector was placed to exteriorize the inflammatory block. The terminal ileum was transected 15 cm proximal to the ileocecal valve, and the ceco-ascending colon was divided just above the pathological segment. An extracorporeal side-to-end ileocolic anastomosis was constructed using a circular stapler (Fig. 2). Peritoneal lavage was performed, hemostasis was verified, and a drain was placed through one of the ports. The abdominal wall at the relaparotomy site was repaired, and four primary-delayed skin sutures were placed with a Betadine-soaked gauze. The procedure was completed without intraoperative complications.

## Postoperative course

**Figure 3:**
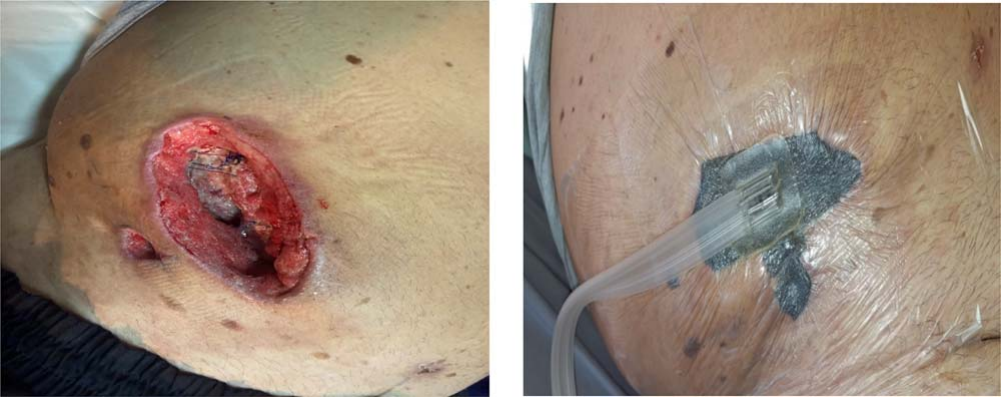
Postoperative wound healing process and NPWT.

On the sixth postoperative day, due to the development of fever, the primary-delayed sutures were removed to allow the wound to heal by secondary intention (per secundam). On the tenth postoperative day, negative pressure wound therapy (NPWT) was applied. The patient was discharged in good general condition with the vacuum system (NPWT) in place (Fig. 3).

## Discussion

Colo-cutaneous fistulae after appendectomy are rare complications, with high-output fistulae presenting significant challenges due to fluid loss, electrolyte imbalance, nutritional depletion, and increased risk of sepsis. Although conservative management, fluid resuscitation, nutritional optimization, and infection control are the initial approaches, high-output fistulae rarely close spontaneously, and delayed surgery may increase morbidity [3]. In this case, laparoscopic revision achieved successful fistula closure with minimal postoperative complications. Laparoscopy offers several advantages, including enhanced visualization for accurate identification of the fistula tract and adhesions, reduced tissue trauma compared to open surgery, and a faster recovery with less postoperative pain. Preoperative imaging, optimization of nutritional and fluid status, and multidisciplinary support are critical to improving outcomes. Intraoperative principles such as careful adhesiolysis and secure closure of the fistula tract further contribute to success [6]. Although published data are limited, existing reports indicate that laparoscopic management of post-appendectomy colo-cutaneous fistulae is a safe and effective option when performed by experienced surgeons. Early operative intervention should be considered in high-output or persistent fistulae to avoid prolonged morbidity [7]. In summary, this case supports the feasibility and efficacy of laparoscopic revision for high-output colo-cutaneous fistulae, offering a minimally invasive route to definitive fistula closure and improved patient recovery.

## Conclusion

High-output colo-cutaneous fistulae following appendectomy are rare but potentially serious complications. While conservative management remains first-line, persistent or high-output fistulae often require surgical intervention. Laparoscopic revision is a safe and effective approach, offering enhanced visualization, reduced adhesion-related trauma, and faster recovery. Successful outcomes depend on careful preoperative planning, multidisciplinary support, and meticulous surgical technique. Early recognition and timely intervention are key to minimizing morbidity and achieving definitive fistula closure.
